# Profitability and risk-return comparison across health care industries, evidence from publicly traded companies 2010–2019

**DOI:** 10.1371/journal.pone.0275245

**Published:** 2022-11-16

**Authors:** Ge Bai, Shivaram Rajgopal, Anup Srivastava, Rong Zhao

**Affiliations:** 1 Johns Hopkins Carey Business School, Baltimore, Maryland, United States of America; 2 Johns Hopkins Bloomberg School of Public Health, Baltimore, Maryland, United States of America; 3 Columbia Business School, New York City, New York, United States of America; 4 Haskayne School of Business, University of Calgary, Calgary, Alberta, Canada; University of Georgia, UNITED STATES

## Abstract

We conducted the first profitability comparison study across health care industries in the United States, using the DuPont Analysis framework. The combination of Return on Equity (ROE) and ROE volatility was used to provide a comprehensive “risk-return” approach for profitability comparison. Based on the 2010–2019 financial disclosures of 1,231 publicly traded health care companies in the U.S. that reported positive assets and equity, we estimated the industry-specific fixed effects on ROE and its three components—profit margin, asset utilization, and financial leverage—for ten industries in the health care sector, classified by the Global Industry Classification Standard (GICS). For each industry, we also estimated its fixed effects on ROE volatility. We found that the pharmaceuticals industry and biotechnology industry have lower ROE—mainly driven by their relatively low profit margin and low assets utilization—and higher ROE volatility than other health care industries. We also found that the health care facilities industry relies most on debt financing. This study demonstrates a holistic approach for profitability comparison across industries.

## Introduction

Comparing profitability across industries is often needed for evidence-based health care policy policymaking. For example, profit margins (profit/revenue) among drug companies, software companies, and companies from other industries have been compared to facilitate the formulation of public policies to address high drug prices and consolidation in the pharmaceuticals industry [1, 2]. Recently, the high profit margin of the health insurance industry during the COVID-19 pandemic, in contrast to the financial distress experienced by some other health care industries, has been cited as a main reason for proposing insurance regulation reforms [3].

Profit margin is useful for intra-industry profitability comparison because companies in the same industry tend to have similar asset utilization efficiency, that is, the ability to turn assets into revenue. However, when it comes to inter-industry comparison, companies in different industries can have widely different asset utilization efficiencies. For example, automobile manufacturers require a much greater asset base than that of software developers to generate the same amount of revenue. Therefore, considering only profit margin ignores the inter-industry variation in the efficiency of asset utilization and provides a narrow perspective [4, 5].

Based on the DuPont Analysis framework, return on equity (ROE; profit/equity) offers a more complete measure of profitability from shareholders’ perspective by combining profit margin, asset utilization, and financial leverage. ROE is a product of profit margin (profit/revenue), asset utilization (revenue/assets), and financial leverage (assets/equity) [6]. For example, in 2019, Pfizer Inc. reported ROE of 0.26–0.31 profit margin multiplied by 0.32 asset utilization and 2.58 financial leverage [7], and UnitedHealth Group reported ROE of 0.25–0.06 profit margin multiplied by 1.49 asset utilization and 2.98 financial leverage [8]. The two companies had similar profitability (ROE: 0.26 vs. 0.25), despite substantial differences in profit margin (0.31 vs. 0.06) and asset utilization (0.32 vs. 1.49).

Moreover, any profitability measure should be considered in conjunction with profit uncertainty or risk, typically measured by volatility [9]. Think of a coin toss that pays 60% of betted amount upon getting a head and requires a payment of 40% upon getting a tail. Any reasonable person would play this game with one thousand tosses with bets of $1 each, calling head each time, hoping to earn $200 profits. But the same person may think twice before playing a game with just one toss with a bet of $1000, because of the high uncertainty of payoff, including the possibility of a $400 loss. Similarly, a person would buy a U.S. government bond and be happy to earn an interest of just 2%, but may be reluctant to invest in a cryptocurrency, which may give higher return, but also carries high risks. Therefore, return on equity (including its three components) along with ROE volatility provides a more complete assessment of risk-return tradeoff and valid comparisons of profitability across different industries [4, 5, 9]. In this study, we do so for U.S. publicly traded companies in health care industries.

## Materials and methods

Any publicly traded company in the world is typically classified into a sector or into an industry in a sector, using the Global Industry Classification Standard (GICS), an industry taxonomy developed by S&P Global and MSCI in 1999 [10]. Audited financial data for all publicly traded companies in the health care sector (two-digit GICS code: 35) were retrieved for fiscal years 2010 to 2019 from Compustat, a database (administered by the Wharton Research Data Services) [11]. These companies are classified into one of 10 health care industries based on their eight-digit GICS codes. Health care equipment (GICS: 35101010) and health care supplies (GICS: 35101020) industries include companies making medical devices or medical supplies and consumables. Health care providers and services subsector includes four industries: health care distributors (GICS: 35102010) that distribute pharmaceuticals and medical products; health care services firms (GICS: 35102015) that primarily provide non-hospital-care services, such as pharmacy, pharmacy benefit management, and kidney dialysis; health care facilities (GICS: 35102020) that operate hospitals and other health care facilities (e.g., nursing homes and physical therapy centers); and managed health care companies (GICS: 35102030) in the health insurance industry. Health care technology firms (GICS: 35103010) are companies that provide technology to support health information and electronic health records. Biotechnology companies (GICS: 35201010) primarily engage in the research, development, manufacturing, and/or marketing of products based on genetic analysis and genetic engineering. Pharmaceuticals (GICS: 35202010) are companies researching, making, and marketing pharmaceutical products. Life sciences tools & services industry (GICS: 35203010) include companies providing scientific instrumentation and data research services. It is worth noting that one publicly traded company can have multiple subsidiaries. For example, Encompass Health Corp has more than 100 rehabilitation hospitals; and HCA Healthcare has almost 200 acute-care hospitals.

For each company-year, we calculate return on equity (ROE = net income/average of beginning and ending book value of equity); profit margin (PM = net income/revenue); asset utilization or asset turnover ratio (ATO = revenue/average of beginning and ending total assets); financial leverage (LEV = average of beginning and ending total assets/average of beginning and ending book value of equity); and market capitalization (stock price × common shares outstanding at the end of the prior fiscal year). For equity and asset, we used the average value in the calculations—consistent with the definitions established in prior literature [4, 5, 9]—to mitigate the measurement noise caused by fluctuations of these balance-sheet accounts across the year. We require the denominator in each ratio calculation to be greater than zero. Starting from 11,622 company-year observations, we exclude 3,755 observations where ROE cannot be calculated, another 1,273 observations where PM, ATO or LEV cannot be calculated. Finally, we exclude 488 observations with missing market capitalization. This sample selection procedure results in 6,106 company-year observations for 1,231 unique health care companies. Every year, many privately held companies choose to become public (through initial public offerings), and many publicly traded companies choose to transition to private ownership, are delisted, or declare bankruptcy. Therefore, in our sample, the number of company-year observations is expected to be smaller than the number of companies multiplied by the number of years. Our data inclusion criteria also restricted the number of company-year observations. We also calculate volatility of overall returns (standard deviation of ROE) for each company over the entire sample period. All continuous variables are winsorized at the 1^st^ and 99^th^ percentile by industry to mitigate the influence of extreme values.

We conduct multivariate regression analyses using the ordinary least squares method, including year fixed effects and industry fixed effects, to compare each performance dimension across 10 health care industries. We create one indicator variable for each industry and include controls for company size (market capitalization) and year fixed effects. We also cluster the standard errors by firm because the regression residuals may be correlated across same-firm observations, which could overstate the significance of our results. To compare the performance of each industry against nine other industries, we use one industry as the benchmark group at a time. Thus, the coefficient on the industry indicator variable represents the comparison between the given industry and the benchmark industry.

## Results

### Descriptive statistics

Table 1 presents the characteristics of the 10 health care industries (ordered by GICS codes with the smallest code on top) during our sample period of 2010–2019. Managed health care industry has the highest median values for each of the four major financial indicators (common shareholders’ equity at $1.5 billion, revenue at $11 billion, net income at $185 million, and total assets at $4.5 billion). The biotechnology industry has the lowest median common equity ($72 million), lowest median revenue ($14 million), lowest net income (-$27 million), and second-lowest median assets ($125 million).

**Table 1:** Characteristics of publicly traded companies in health care industries, in GICS order.

Industry (Eight-digit GICS)		# of company-years (companies)	Top 3 companies (by 2019 revenue)	Median book equity $ millions (IQR)	Median revenue $ millions (IQR)	Median profit $ millions (IQR)	Median assets $ millions (IQR)
(1)	Health care equipment (35101010)	1,226	Abbott Laboratories	$78	$82	-$1	$119
		(219)	Medtronic PLC	($11, $481)	($13, $541)	(-$14, $25)	($21, $821)
			Koninklijke Philips NV				
(2)	Health care supplies (35101020)	347	Dentsply Sirona Inc	$98	$113	-$1	$156
		(59)	Cooper Cos Inc	($31, $465)	($34, $379)	(-$17, $29)	($53, $633)
			Align Technology Inc				
(3)	Health care distributors (35102010)	108	McKesson Corp	$939	$5,426	$74	$2,699
		(22)	AmerisourceBergen Corp	($240, $2,840)	($553, $84,088)	($6, $450)	($479, $17,181)
			Cardinal Health Inc				
(4)	Health care services (35102015)	501	CVS Health Corp	$163	$426	$9	$388
		(93)	Cigna Corp	($26, $515)	($60, $1,624)	(-$3, $65)	($64, $1,166)
			Fresenius Medical Care				
(5)	Health care facilities (35102020)	239	Universal Health Services Inc	$289	$1,041	$5	$1,081
		(45)	Select Medical Holdings Corp	($42, $978)	($257, $3,359)	(-$14, $92)	($224, $4,722)
			Encompass Health Corp				
(6)	Managed health care (35102030)	119	UnitedHealth Group Inc	$1,557	$11,901	$185	$4,477
		(17)	Anthem Inc	($863, $10,406)	($2,996, $54,289)	($54, $1,683)	($2,102, $30,901)
			Centene Corp				
(7)	Health care technology (35103010)	227	Cerner Corp	$126	$173	$0	$249
		(50)	Allscripts Healthcare Solutions	($15, $463)	($23, $492)	(-$9, $20)	($32, $883)
			Veena Systems Inc				
(8)	Biotechnology (35201010)	1,937	Amgen Inc	$72	$14	-$27	$125
		(448)	Gilead Sciences Inc	($19, $226)	($2, $76)	(-$69, -$6)	($42, $367)
			Biogen Inc				
(9)	Pharmaceuticals (35202010)	1,017	Johnson & Johnson	$88	$49	-$5	$164
		(214)	Roche Holding AG	($18, $888)	($7, $880)	(-$35, $65)	($49, $2,341)
			Pfizer Inc				
(10)	Life sciences tools & services (35203010)	385	Thermo Fisher Scientific Inc	$249	$247	$10	$438
		(64)	IQVIA Holdings Inc	($25, $1,046)	($33, $1,839)	(-$5, $125)	($53, $2,288)
			Agilent Technologies Inc				
	**All Industries**	**6,106**	**CVS Health Corp**	**$100**	**$65**	**-$4**	**$174**
		**(1,231)**	**UnitedHealth Group Inc**	**($19, $512)**	**($9, $636)**	**(-$32, $29)**	**($42, $980)**
			**McKesson Corp**				

**Notes:** Return on Equity (ROE), Profit Margin (PM), Asset Utilization (ATO) and Financial Leverage (LEV) are calculated for each company in each fiscal year. The total sample is comprised of 6,106 company-years of data from 2010 to 2019. Return on Equity is net income divided by the average of beginning and ending book value of equity. Profit Margin is net income divided by revenue. Asset Utilization is revenue divided by the average of beginning and ending total assets. Financial Leverage is the average of beginning and ending total assets divided by the average of beginning and ending book value of common equity. ROE Volatility (ROE_VOL) is the standard deviation of annual ROEs for each company during 2010–2019.

As shown by the summary statistics in Table 2, median ROE and median profit margin for the health care sector as a whole are negative (-12.6% and -8.9%, respectively). Both ROE and profit margin are skewed to the left as mean values are much lower than the median values. Median asset utilization ratio is 0.532, indicating that the median health care firm generates $0.53 of sales for every dollar of total assets. Median leverage of 1.735 indicates that total assets is 1.735 times of total common equity (or approximately 57.6% of total assets are financed by common equity). ROE volatility or standard deviation of ROE is skewed to the right with median value of 0.273 and mean of 0.953.

**Table 2:** Summary statistics.

Variable	N	Mean	Standard Deviation	1st Quartile	Median	3rd Quartile
ROE	6,106	-0.723	2.225	-0.725	-0.126	0.112
PM	6,106	-22.335	125.825	-2.308	-0.089	0.068
ATO	6,106	0.669	0.691	0.180	0.532	0.886
LEV	6,106	3.077	9.841	1.312	1.735	2.568
ROE_VOL	1,024	0.953	1.743	0.093	0.273	0.875

**Notes:** Return on Equity (ROE), Profit Margin (PM), Asset Utilization (ATO) and Financial Leverage (LEV) are calculated for each company in each fiscal year. The total sample is comprised of 6,106 company-years of data from 2010 to 2019. Return on Equity is net income divided by the average of beginning and ending book value of equity. Profit Margin is net income divided by revenue. Asset Utilization is revenue divided by the average of beginning and ending total assets. Financial Leverage is the average of beginning and ending total assets divided by the average of beginning and ending book value of common equity. ROE Volatility (ROE_VOL) is the standard deviation of annual ROEs for each company during 2010–2019.

**Fig 1:**
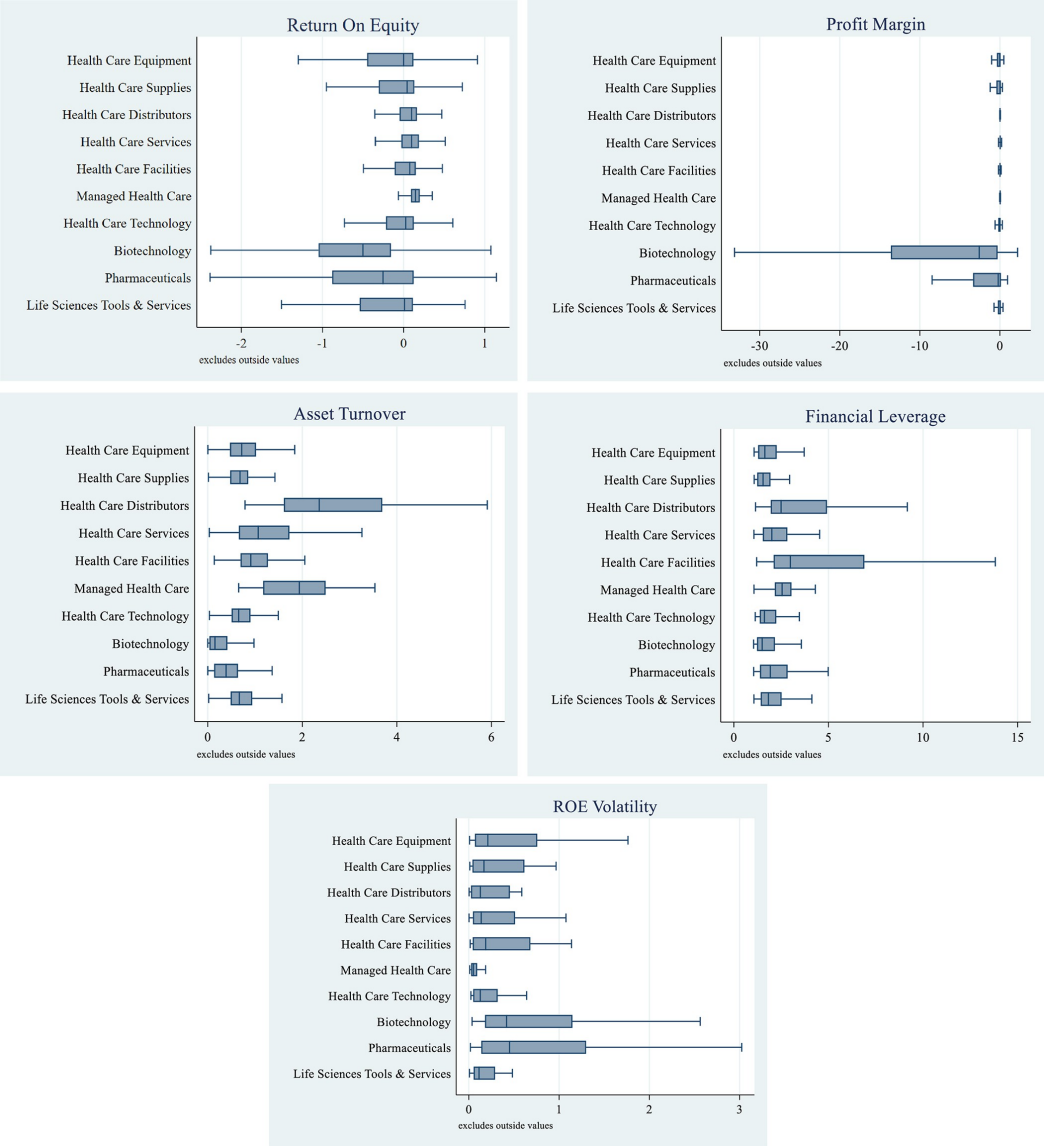
Firm performance across health care industries. A: Return on Equity (ROE), B: Profit Margin, C: Asset Utilization, D: Financial Leverage, E: ROE Volatility. **Notes:** Box plot lines represent the 25th percentile, median, and 75th percentile. Whiskers are 1.5 times the interquartile range. Return on Equity (ROE), Profit Margin (PM), Asset Utilization (ATO) and Financial Leverage (LEV) are calculated for each company in each fiscal year. The total sample is comprised of 6,106 company-years of data from 2010 to 2019. Return on Equity is net income divided by the average of beginning and ending book value of equity. Profit Margin is net income divided by revenue. Asset Utilization is revenue divided by the average of beginning and ending total assets. Financial Leverage is the average of beginning and ending total assets divided by the average of beginning and ending book value of equity. ROE Volatility (ROE_VOL) is the standard deviation of annual ROEs for each company during 2010–2019.

Box and whisker plots for the distribution of each variable by industry are presented in Fig 1. The biotechnology and pharmaceuticals industries have the widest interquartile range for ROE (Panel A), profit margin (Panel B), and ROE volatility (Panel E). Panel A also shows that the ROE interquartile ranges of the four industries within the health care providers and services subsector−health care distributors, health care services, health care facilities, and managed health care−are the smallest among the 10 industries. The remaining industries−health care equipment, health care supplies, health care technology, and life sciences tools & services−have ROE interquartile ranges that are in the middle. An examination of ROE volatility in Panel E shows that the managed health care, life sciences tools & services, and health care technology industries have the smallest interquartile range for ROE volatility.

### Regression results

The following key messages emerge from the regression results in Table 3.

**Table 3:** Regression analysis results.

**Panel A: Return on Equity (ROE)**									
	**Reference Industry**								
Industry	(1)	(2)	(3)	(4)	(5)	(6)	(7)	(8)	(9)
(1) Health Care Equipment									
(2) Health Care Supplies	-0.193								
	[-0.82]								
(3) Health Care Distributors	0.421***	0.613**							
	[2.93]	[2.38]							
(4) Health Care Services	0.368***	0.560**	-0.053						
	[4.13]	[2.41]	[-0.37]						
(5) Health Care Facilities	0.275**	0.468*	-0.146	-0.093					
	[2.11]	[1.86]	[-0.86]	[-0.73]					
(6) Managed Health Care	0.487***	0.679***	0.066	0.119	0.212				
	[4.41]	[2.81]	[0.42]	[1.10]	[1.48]				
(7) Health Care Technology	0.260**	0.453*	-0.161	-0.108	-0.015	-0.227*			
	[2.17]	[1.84]	[-0.99]	[-0.92]	[-0.10]	[-1.69]			
(8) Biotechnology	-0.614***	-0.421*	-1.034***	-0.981***	-0.889***	-1.100***	-0.873***		
	[-5.52]	[-1.73]	[-6.61]	[-9.11]	[-6.17]	[-8.71]	[-6.54]		
(9) Pharmaceuticals	-0.425***	-0.232	-0.846***	-0.793***	-0.700***	-0.912***	-0.685***	0.189	
	[-3.45]	[-0.93]	[-5.17]	[-6.60]	[-4.54]	[-6.75]	[-4.70]	[1.36]	
(10) Life Sciences Tools & Services	0.126	0.319	-0.294	-0.241*	-0.149	-0.360**	-0.134	0.740***	0.551***
	[0.85]	[1.22]	[-1.59]	[-1.65]	[-0.85]	[-2.24]	[-0.80]	[4.59]	[3.25]
**Panel B: Profit Margin (PM)**									
	**Reference Industry**								
Industry	(1)	(2)	(3)	(4)	(5)	(6)	(7)	(8)	(9)
(1) Health Care Equipment									
(2) Health Care Supplies	4.438***								
	[2.59]								
(3) Health Care Distributors	4.282**	-0.156							
	[2.45]	[-0.13]							
(4) Health Care Services	4.789***	0.351	0.507						
	[3.00]	[0.37]	[0.50]						
(5) Health Care Facilities	5.439***	1.001	1.157	0.650					
	[3.33]	[1.05]	[1.10]	[0.89]					
(6) Managed Health Care	1.841	-2.597	-2.441	-2.948	-3.598				
	[0.68]	[-1.08]	[-1.02]	[-1.28]	[-1.56]				
(7) Health Care Technology	4.677***	0.239	0.394	-0.112	-0.762	2.836			
	[2.66]	[0.20]	[0.30]	[-0.11]	[-0.71]	[1.16]			
(8) Biotechnology	-46.062***	-50.500***	-50.345***	-50.851***	-51.501***	-47.903***	-50.739***		
	[-6.65]	[-7.43]	[-7.49]	[-7.56]	[-7.69]	[-6.90]	[-7.44]		
(9) Pharmaceuticals	-21.111***	-25.549***	-25.393***	-25.900***	-26.550***	-22.952***	-25.788***	24.951***	
	[-3.84]	[-4.70]	[-4.85]	[-4.88]	[-4.93]	[-4.25]	[-4.74]	[2.90]	
(10) Life Sciences Tools & Services	4.647***	0.209	0.365	-0.142	-0.792	2.806	-0.030	50.709***	25.758***
	[2.91]	[0.22]	[0.36]	[-0.20]	[-1.10]	[1.22]	[-0.03]	[7.55]	[4.85]
**Panel C: Asset Utilization (ATO)**									
	**Reference Industry**								
Industry	(1)	(2)	(3)	(4)	(5)	(6)	(7)	(8)	(9)
(1) Health Care Equipment									
(2) Health Care Supplies	-0.065								
	[-1.14]								
(3) Health Care Distributors	2.042***	2.107***							
	[5.60]	[5.76]							
(4) Health Care Services	0.457***	0.522***	-1.585***						
	[5.01]	[5.41]	[-4.25]						
(5) Health Care Facilities	0.303**	0.367***	-1.739***	-0.155					
	[2.52]	[2.96]	[-4.57]	[-1.08]					
(6) Managed Health Care	1.084***	1.148***	-0.958**	0.626***	0.781***				
	[5.56]	[5.81]	[-2.33]	[2.98]	[3.48]				
(7) Health Care Technology	0.059	0.124	-1.983***	-0.398***	-0.244	-1.025***			
	[0.50]	[1.02]	[-5.22]	[-2.82]	[-1.51]	[-4.60]			
(8) Biotechnology	-0.493***	-0.429***	-2.535***	-0.951***	-0.796***	-1.577***	-0.552***		
	[-13.33]	[-8.91]	[-6.98]	[-11.05]	[-6.84]	[-8.18]	[-4.89]		
(9) Pharmaceuticals	-0.344***	-0.280***	-2.386***	-0.802***	-0.647***	-1.428***	-0.403***	0.149***	
	[-8.61]	[-5.49]	[-6.56]	[-9.14]	[-5.52]	[-7.42]	[-3.53]	[5.59]	
(10) Life Sciences Tools & Services	-0.027	0.038	-2.069***	-0.484***	-0.330***	-1.111***	-0.086	0.466***	0.317***
	[-0.46]	[0.57]	[-5.65]	[-4.95]	[-2.64]	[-5.60]	[-0.70]	[9.16]	[5.95]
**Panel D: Leverage (LEV)**									
	**Reference Industry**								
Industry	(1)	(2)	(3)	(4)	(5)	(6)	(7)	(8)	(9)
(1) Health Care Equipment									
(2) Health Care Supplies	0.413								
	[0.78]								
(3) Health Care Distributors	2.003**	1.590							
	[2.31]	[1.58]							
(4) Health Care Services	1.454***	1.042	-0.548						
	[2.83]	[1.43]	[-0.55]						
(5) Health Care Facilities	9.647***	9.235***	7.644**	8.193***					
	[3.08]	[2.91]	[2.36]	[2.59]					
(6) Managed Health Care	0.448**	0.035	-1.555*	-1.006*	-9.199***				
	[2.07]	[0.06]	[-1.76]	[-1.87]	[-2.94]				
(7) Health Care Technology	0.037	-0.375	-1.966**	-1.417**	-9.610***	-0.411			
	[0.15]	[-0.66]	[-2.20]	[-2.52]	[-3.06]	[-1.32]			
(8) Biotechnology	0.326**	-0.086	-1.677*	-1.128**	-9.321***	-0.122	0.289		
	[2.12]	[-0.16]	[-1.92]	[-2.15]	[-2.96]	[-0.51]	[1.08]		
(9) Pharmaceuticals	1.011***	0.598	-0.992	-0.443	-8.636***	0.563*	0.974***	0.685***	
	[4.14]	[1.05]	[-1.11]	[-0.80]	[-2.75]	[1.91]	[2.94]	[2.63]	
(10) Life Sciences Tools & Services	0.370	-0.043	-1.633*	-1.084*	-9.277***	-0.078	0.333	0.044	-0.641*
	[1.29]	[-0.07]	[-1.81]	[-1.89]	[-2.96]	[-0.23]	[0.91]	[0.14]	[-1.81]
**Panel E: ROE Volatility (ROE_VOL)**									
	**Reference Industry**								
Industry	(1)	(2)	(3)	(4)	(5)	(6)	(7)	(8)	(9)
(1) Health Care Equipment									
(2) Health Care Supplies	0.517*								
	[1.92]								
(3) Health Care Distributors	-0.211	-0.728							
	[-0.51]	[-1.58]							
(4) Health Care Services	-0.315	-0.833***	-0.104						
	[-1.36]	[-2.69]	[-0.24]						
(5) Health Care Facilities	-0.152	-0.669*	0.059	0.163					
	[-0.49]	[-1.81]	[0.12]	[0.48]					
(6) Managed Health Care	-0.541	-1.059**	-0.330	-0.226	-0.389				
	[-1.18]	[-2.10]	[-0.56]	[-0.47]	[-0.74]				
(7) Health Care Technology	-0.404	-0.921**	-0.193	-0.089	-0.252	0.138			
	[-1.30]	[-2.47]	[-0.40]	[-0.26]	[-0.63]	[0.26]			
(8) Biotechnology	0.522***	0.005	0.733*	0.837***	0.674**	1.063**	0.926***		
	[3.43]	[0.02]	[1.82]	[3.88]	[2.28]	[2.35]	[3.10]		
(9) Pharmaceuticals	0.409**	-0.109	0.619	0.724***	0.561*	0.950**	0.812***	-0.114	
	[2.27]	[-0.40]	[1.50]	[3.07]	[1.80]	[2.06]	[2.58]	[-0.71]	
(10) Life Sciences Tools & Services	-0.036	-0.554*	0.174	0.279	0.116	0.505	0.367	-0.559**	-0.445*
	[-0.14]	[-1.66]	[0.38]	[0.92]	[0.32]	[1.01]	[1.00]	[-2.24]	[-1.67]

**Notes:** Differentials between industries are estimated using the multivariate regression models including an indicator variable for each industry, a control for company size (market capitalization), and year fixed effects to control for time trends (year fixed effects are omitted in the regression for ROE Volatility). Model (1) uses industry (1) health care equipment as the reference group, model (2) uses industry (2) health care supplies as the reference group, and so on. This table presents coefficients on the industry indicator variables where each coefficient represents whether a given industry is significantly different from the reference industry along the performance dimension examined. A positive/negative value indicates that the value for that industry is larger/smaller than that of the reference group. The coefficients above the diagonal are omitted for brevity. The regression sample for ROE, PM, ATO and LEV is comprised of 6,106 company-years from 2010 to 2019. The regression sample for ROE_VOL is comprised of 1,024 companies. The numbers above brackets present estimated coefficients. *t*-statistics are presented in brackets.

*, **, and *** denote significance at the 0.10, 0.05, and 0.01 level, respectively, on a two-tailed basis. The adjusted R squared are 0.05, 0.04, 0.45, 0.03, and 0.04 for ROE, PM, ATO, LEV and ROE_VOL models in Panels A, B, C, D, and E, respectively. Untabulated results on year fixed effects show that in general, ROE, PM, and ATO had been declining over our sample period whereas LEV had been inching upward (except for 2013–2014).

### ROE and DuPont analysis

We start with the overall return by comparing ROE in Panel A. Biotechnology and pharmaceuticals industries (industries 8 and 9) have the lowest ROE. Life sciences tools & services, health care equipment, and health care supplies industries (industries 10, 1 and 2) are in the middle. Health care technology (industry 7) and four industries within the health care providers and services subsector (health care distributors, health care services, health care facilities, and managed health care, or industries 3, 4, 5 and 6) have the highest ROE.

Next, we evaluate each component of the DuPont decomposition for ROE. Results in Panel B and Panel C show that biotechnology and pharmaceuticals industries again have significantly lower profit margin and lower asset utilization than the remaining industries. Thus, the low ROE for these two industries is driven by both low profit margin and low asset utilization. The remaining industries have similar levels of profit margin and asset utilization except the health care equipment companies (industry 1) whose profit margin and asset utilization ratios are in general low. In terms of financial leverage, the health care facilities industry (industry 5) stands out as the one industry that consistently show higher financial leverage than the rest (Panel D).

### ROE volatility

Panel E shows that biotechnology and pharmaceuticals industries (industries 8 and 9) consistently have the highest ROE volatility. The rest of the industries have similar risk profiles except the health care supplies (industry 2) whose ROE volatility is significantly higher than most of the other industries.

### Limitations

This study is confined to publicly traded companies in the U.S. The number of publicly traded companies varies substantially across health care industries, reflecting each industry’s idiosyncratic characteristics. However, to the extent that the results may not be generalizable to privately held companies or organizations of different ownership types, the data’s lack of representativeness in some industries is an important limitation. This limitation is especially relevant for industries dominated by such organizations—for example, nonprofit organizations play a critical role in the health care facilities industry in the U.S. Moreover, the results, based on data from 2010 and 2019, cannot be generalized to previous or subsequent periods. In addition, this study, limited by potential omitted-variable biases, cannot provide evidence to support any causal relationship. Finally, the GICS industry classification is subject to measurement noise due to the diversity of business operations of many large health care companies.

## Discussion and conclusion

In this study, we conducted the first profitability comparison study, based on the Dupont Analysis framework, across health care industries in the United States using data from publicly traded companies between 2010 and 2019. The combination of ROE (including its three components: profit margin, assets utilization, and financial leverage) and ROE volatility provides a comprehensive “risk-return” approach to facilitate inter-industry profitability comparison. Using this approach, we found that the pharmaceuticals industry and biotechnology industry have lower ROE—mainly driven by their relatively low profit margin and low asset utilization—and higher ROE volatility than other health care industries. These two industries demonstrated a low-return-high-risk profile in comparison to other health care industries. We also found that the health care facilities industry relies most on debt financing.

This study aims to demonstrate a holistic approach for profitability comparison across industries, which is often used by policymakers to formulate evidence-based health care policies. Profitability comparison should focus on the combination of risk and return, namely, ROE (and its components: asset utilization, profit margin, and financial leverage) and ROE volatility. Solely relying on profit margin to compare profitability across industries ignores the difference in how efficiently these industries utilize their assets and the differences in their profit volatility, and thus may not provide a complete assessment. It is worth emphasizing that our results are based on 10 years of data (2010–2019) for publicly traded companies in health care industries. Caution is needed in extrapolating the results to a specific subset of companies, organizations that are not publicly traded, or to a different time period.
